# High efficacy of combination chemotherapy with S-1 and low-dose docetaxel for the treatment of highly advanced gastric cancer with peritoneal dissemination: A case report

**DOI:** 10.3892/ol.2013.1237

**Published:** 2013-03-08

**Authors:** LUCHENG ZHU, WENHUA LUO, JUAN WEI, CHANGLIN ZOU

**Affiliations:** Department of Radio-Chemotherapy Oncology, The First Affiliated Hospital of Wenzhou Medical College, Wenzhou 325035, P.R. China

**Keywords:** gastric cancer, chemotherapy, docetaxel, S-1, peritoneal metastasis, peritoneal dissemination

## Abstract

S-1 has been developed as a new oral anticancer drug based on the biological modulation of 5-fluorouracil. We treated a patient with highly advanced gastric carcinoma with a new combination chemotherapy of S-1 and low-dose docetaxel. Evident tumor reduction was observed following two cycles of this therapy in the primary tumor and metastatic lymph nodes. This was concluded to be a partial response. The gastric tumor exhibited significant invasion to the serosa. Lymph nodes around the stomach were swollen. Peritoneal dissemination was also identified in the mesocolon. Palliative resection of part of the distal stomach and dissection of regional lymph nodes were performed. Histological examination demonstrated that no tumor cells were detected in the greater omentum, suggesting pathological complete remission. It is suggested that this regimen may provide a potent combined therapy for the treatment of patients with highly advanced gastric carcinoma and it may be useful as an adjuvant chemotherapy. Further studies are necessary to evaluate the efficacy of this therapy.

## Introduction

Peritoneal metastasis, a common type of metastasis in advanced gastric cancer, causes several complications, including ascites, bowel obstruction and hydronephrosis, all leading to a deterioration of the patient’s general condition (1). In the present study, we report a case of using S-1 plus docetaxel to treat a patient with peritoneal metastasis, demonstrating that this therapy is well-tolerated and has high efficacy even when a patient has peritoneal metastasis. The study was approved by the Ethics Committee of The First Affiliated Hospital of Wenzhou Medical College, Wenzhou, China.

## Case report

### Clinical presentation and diagnosis

A 64-year-old male was admitted to The First Affiliated Hospital of Wenzhou Medical College presenting with an epigastric mass. A mass of 1×1 cm was identified in the epigastric region following a physical examination. The serum levels of CEA, CA125, CA19-9 and CA724 were 16.1 *μ*g/l (normal range, <5 *μ*g/l), 47.0 U/ml (normal range, <35 U/ml), 1151.7 U/ml (normal range, <37 U/ml) and 224.2 U/ml (normal range, <3 U/ml), respectively. Other data obtained from laboratory investigations were within the normal ranges.

**Figure 1:**
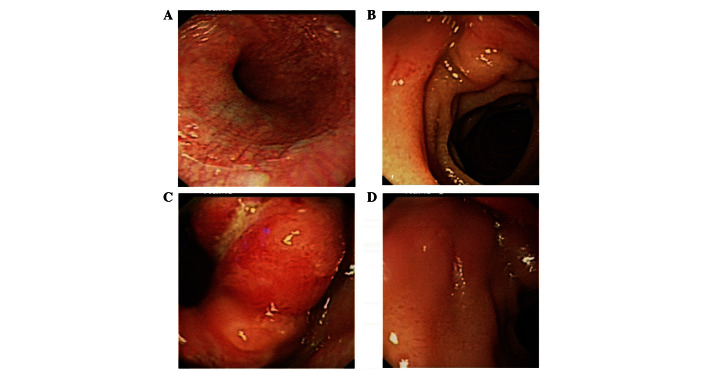
Endoscopic examination prior to chemotherapy.

**Figure 2:**
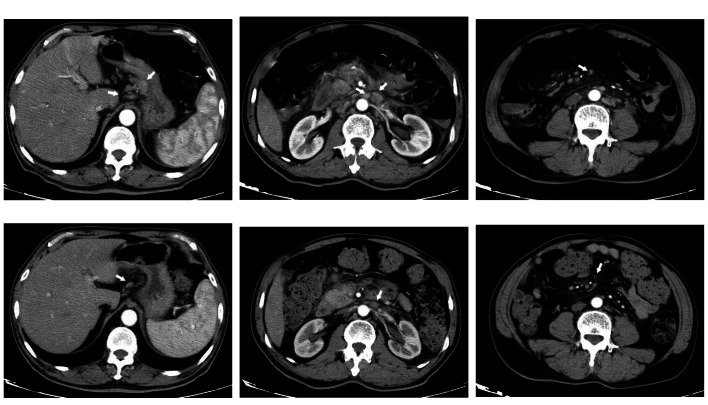
CT examination (A–C) prior to chemotherapy and (D–F) following two cycles of chemotherapy. Arrows show metastatic lymph nodes. CT, computed tomography.

Gastrointestinal endoscopy (Fig. 1) revealed gastric carcinoma of the gastricum. Endoscopic biopsy specimens were collected from the gastric lesions and then fixed and stained with hematoxylin and eosin. Histological examination of the biopsy specimens revealed adenocarcinoma. Abdominal computed tomography (CT) scan demonstrated a thickening of the gastric sinus wall and enlargement of lymph nodes around the stomach and peritoneum, and also in the para-aortal region (Fig. 2A–C). Both were notably enhanced when contrast material was injected, suggesting large lymph node metastases. These results suggest that the gastric tumor identified in this patient was highly advanced, corresponding to stage IV of the UICC classification, and therefore, curative surgery was not a suitable treatment option.

### Treatment schedules

S-1 was administered orally at a dose of 80 mg/m^2^/day on days 1–14 and docetaxel was infused at a dose of 40 mg/m^2^/day on day 1. This treatment regimen was repeated every 3 weeks for two cycles and following this, the patient was evaluated for response. In advance of this therapy, written informed consent was obtained from the patient and the patients’ families.

### Objective response and toxicities

The patient’s response was evaluated using CT following two cycles of treatment. CT demonstrated that the lymph nodes around the stomach and peritoneum notably decreased in size (Fig. 2D–F). The objective response was evaluated as a partial response in the gastric tumor and metastatic lymph nodes according to the World Health Organization (WHO) criteria (2). The serum levels of CA125 were normalized and others were significantly decreased.

No significant adverse effects were observed. No gastrointestinal or bone marrow toxicities were detected. The patient maintained a good quality of life and performance status remained at grade 0 according to the WHO grading during chemotherapy.

### Surgery and pathological results

A gastric tumor was identified on the gastricum, demonstrating an evident invasion to the serosa. Lymph nodes surrounding the stomach, particularly around the lesser curvature of the stomach, were evidently swollen. However, para-aortal lymph node swelling was not recognized. Peritoneal dissemination was also recognized in the omentum and mesocolon. Palliative resection of part of the distal stomach and dissection of regional lymph nodes were performed.

**Figure 3:**
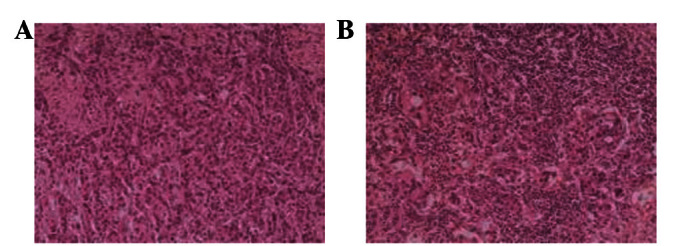
Postoperative pathological results demonstrated that the lesion was poorly differentiated (hematoxylin and eosin staining; magnification, ×200).

Postoperative pathological results (Fig. 3) demonstrated a poorly differentiated carcinoma on the lesser curvature of the stomach with a size of 3×2 cm, pT4aN3bM0 with serosa invasion. Both nerve and vascular invasion were evident. A total of 19 local lymph nodes metastases were detected in 19 retrieved lymph nodes, with perineurovascular invasion; however, no tumor cells were detected in the greater omentum.

### Postoperative course

The patient has maintained good health since surgery. Another cycle of low-dose docetaxel and S-1 combination chemotherapy is planned to be administered as adjuvant therapy. The patient will be continually monitored.

## Discussion

In advanced gastric cancer, peritoneal dissemination is the most frequent site of distant metastasis. The prognosis of patients diagnosed with peritoneal dissemination is extremely poor. Therefore, the development of an effective therapy against peritoneal dissemination is urgently needed. To date, several clinical trials with single-agent chemotherapy or combination chemotherapy have been performed and used to guide the treatment of advanced gastric cancer. The response rate to single-agent chemotherapy was reported to be 15–30% (2–4), and with a number of drug combinations, improved to 40–73% (5–8). However, toxicity was increased in combinational chemotherapy.

S-1 is a novel oral fluoropyrimidine derivative consisting of tegafur (FT), 5-chloro-2,4-dihydroxypyridine (CDHP) and potassium oxonate (Oxo) at a molar ratio of 1:0.4:1, respectively. FT is a prodrug of 5-fluorouracil (5-FU) and is gradually converted to 5-FU by the cytochrome p450 enzyme in the liver. CDHP is a potent inhibitor of dihydropyrimidine dehydrogenase (DPD). Gastrointestinal (GI) toxicities, including diarrhea and mucositis, are dose-limiting factors associated with the use of 5-FU. Oxo is a reversible competitive inhibitor of orotate phosphoribosy1 transferase (OPRT), an enzyme that is responsible for GI toxicity via its phosphorylation of 5-FU. Therefore, the oral administration of S-1 is capable of achieving a more potent antitumor effect through an increased 5-FU concentration without any enhancement of GI toxicity. S-1 has recently been adopted as a front-line chemotherapeutic regimen for the treatment of gastric cancer in Japan, based on the results of several randomized trials (9,10).

Peritoneal dissemination is the most frequent cause of mortality from gastric cancer. However, systemic chemotherapy has a poor response rate and has been attributed to the inadequate passage of systemically administered anticancer drugs to the peritoneal tissue (11) as well as the prompt absorption of intraperitoneally administered drugs from the peritoneum, resulting in low drug concentrations in the peritoneal cavity (12). Sugarbaker *et al* ascribed the poor therapeutic response of peritoneal dissemination to systemic chemotherapy to the blood-peritoneal barrier, consisting of the endothelium, mesentery and intervening stromal tissue, which separates the cardiovascular system from the peritoneal cavity (11). This barrier negatively affects drug delivery via the bloodstream in patients receiving systemic chemotherapy. Following the administration of S-1 to gastric cancer patients with peritoneal dissemination, 5-FU and CDHP linearly pass through the peritoneum and enter the ascites in serum. High concentrations of 5-FU selectively penetrate disseminated peritoneal cells (13). Another study demonstrated that S-1 exhibits a high rate of transfer into the peritoneal metastasis (14). This may explain the positive effects of chemotherapy containing S-1 to peritoneal metastasis.

Docetaxel, a semisynthetic taxane, disrupts microtubule dynamics by stabilizing the microtubules against depolymerization. It has been tested and identified to be active against advanced gastric cancer. It has shown promising activity in gastric cancer as a monotherapy and in combination with other agents.

Docetaxel and S-1 have different modes of action and are highly synergistic in gastric cancer xenografts. Also, the synergistic effects of docetaxel and S-1 were evident *in vivo* and *in vitro* and may be explained by docetaxel’s biochemical modulation of the expression and activity of the thymidylate synthase (TS), DPD and OPRT enzymes, which are important in the functional activities of 5-FU or S-1. The docetaxel/S-1 combination was active and well tolerated in a phase I, a phase I/II and a phase II study in advanced gastric cancer, although the treatment schedules were slightly different. The result of a recent phase II study in patients with advanced gastric cancer (docetaxel 40 mg/m^2^ administered intravenously over 1 h on day 1 and oral S-1 administered at a fixed dose of 40 mg/m^2^ twice daily on days 1–14 of each 3-week cycle) was highly promising (a response rate of 56.3% with a median survival time of 14.3 months), with tolerable toxicities (15); accordingly, a multinational randomized phase III study comparing S-1 alone versus docetaxel plus S-1 is ongoing at this dose and schedule. A relatively low dose of docetaxel was recommended in this study, possibly as a result of the conservative definitions used for dose-limiting toxicities (DLTs) in the previous phase I study (i.e., the DLTs were mild compared with the definitions used in other phase I studies) (16). This resulted in the need to conduct a further dose-finding phase I trial of the docetaxel and S-1 combination.

In conclusion, we have reported a new combined chemotherapy of S-1 with low-dose docetaxel, suggesting that it may be an extremely potent regimen without any serious adverse effects for patients with advanced gastric cancer with peritoneal metastasis. Further studies are required to evaluate the efficacy of this therapy.
